# Esmolol for cardioprotection during resuscitation with adrenaline in an ischaemic porcine cardiac arrest model

**DOI:** 10.1186/s40635-019-0279-5

**Published:** 2019-12-04

**Authors:** Hilde Karlsen, Harald Arne Bergan, Per Steinar Halvorsen, Kjetil Sunde, Eirik Qvigstad, Geir Øystein Andersen, Jan Frederik Bugge, Theresa Mariero Olasveengen

**Affiliations:** 1 Department of Research and Development, Oslo University Hospital, PB 4956 Nydalen, N-0424 Oslo, Norway; 2 The Intervention Center, Department of Anaesthesiology, Oslo University Hospital, Oslo, Norway; 3 Department of Anaesthesiology, Oslo University Hospital, Oslo, Norway; 4 Institute of Clinical Medicine, University of Oslo, Oslo, Norway; 5 Department of Cardiology, Oslo University Hospital, Oslo, Norway; 6 Oslo Cardiopulmonary Resuscitation Research Network, Oslo, Norway

**Keywords:** Resuscitation, Cardiopulmonary resuscitation, Esmolol, β-adrenergic blocker, Extra-corporal membrane oxygenation (ECMO), Porcine model, Myocardial infarction, Cardiac MRI, Cardioprotection

## Abstract

**Background:**

The effectiveness of adrenaline during resuscitation continues to be debated despite being recommended in international guidelines. There is evidence that the β-adrenergic receptor (AR) effects of adrenaline are harmful due to increased myocardial oxygen consumption, post-defibrillation ventricular arrhythmias and increased severity of post-arrest myocardial dysfunction. Esmolol may counteract these unfavourable β-AR effects and thus preserve post-arrest myocardial function. We evaluated whether a single dose of esmolol administered prior to adrenaline preserves post-arrest cardiac output among successfully resuscitated animals in a novel, ischaemic cardiac arrest porcine model.

**Methods:**

Myocardial infarction was induced in 20 anaesthetized pigs by inflating a percutaneous coronary intervention (PCI) balloon in the circumflex artery 15 min prior to induction of ventricular fibrillation. After 10 min of untreated VF, resuscitation with veno-arterial extracorporeal membrane oxygenation (VA-ECMO) was initiated and the animals were randomized to receive an injection of either 1 mg/kg esmolol or 9 mg/ml NaCl, prior to adrenaline. Investigators were blinded to allocation. Successful defibrillation was followed by a 1-h high-flow VA-ECMO before weaning and an additional 1-h stabilization period. The PCI-balloon was deflated 40 min after inflation. Cardiac function pre- and post-arrest (including cardiac output) was assessed by magnetic resonance imaging (MRI) and invasive pressure measurements. Myocardial injury was estimated with MRI, triphenyl tetrazolium chloride (TTC) staining and serum concentrations of cardiac troponin T.

**Results:**

Only seven esmolol and five placebo-treated pigs were successfully resuscitated and available for post-arrest measurements (*p* = 0.7). MRI revealed severe but similar reductions in post-arrest cardiac function with cardiac output 3.5 (3.3, 3.7) and 3.3 (3.2, 3.9) l/min for esmolol and control (placebo) groups, respectively (*p* = 0.7). The control group had larger left ventricular end-systolic and end-diastolic ventricular volumes compared to the esmolol group (75 (65, 100) vs. 62 (53, 70) ml, *p* = 0.03 and 103 (86, 124) vs. 87 (72, 91) ml, *p* = 0.03 for control and esmolol groups, respectively). There were no other significant differences in MRI characteristics, myocardial infarct size or other haemodynamic measurements between the two groups.

**Conclusions:**

We observed similar post-arrest cardiac output with and without a single dose of esmolol prior to adrenaline administration during low-flow VA-ECMO in an ischaemic cardiac arrest pig model.

**Electronic Supplementary Information:**

Supplementary material is available for this article at 10.1186/s40635-019-0279-5 and is accessible for authorized users.

## Background

Cardiac arrest remains a major public health problem with an estimated 100 per 100,000 Europeans affected every year. Although cardiac arrest management and training are constantly evolving, only on average 10% of patients who suffer out-of-hospital cardiac arrest (OHCA) can currently be expected to survive the event [1]. Current initial treatment for cardiac arrest involves high-quality chest compressions, ventilations, and timely defibrillations for those with shockable rhythms [2]. Drugs like adrenaline, amiodarone and to some extent lidocaine are also recommended in current European treatment guidelines [3], but their efficiency remains heavily debated. There is an urgent need for more effective drugs to improve both initial resuscitation and long-term outcomes after cardiac arrest.

Adrenaline has potent vasoconstrictive α-adrenergic receptor (AR) effects that have been consistently shown to increase coronary perfusion pressure (CPP) and return of spontaneous circulation (ROSC) in laboratory studies [4–6]. Randomized controlled trials on adrenaline have confirmed the drug’s ability to improve ROSC rates, but have largely failed to translate the large improvements in ROSC into long-term survival with good neurological outcome [7–9]. It is questioned whether the coveted α-AR effects are negated by unwanted β–AR effects, such as increased myocardial oxygen consumption [10], post-arrest ventricular arrhythmias [11, 12] and post-arrest myocardial dysfunction [13, 14]. Counteracting β–AR stimulation with a β–AR antagonist has been shown to be cardioprotective, anti-arrhythmic and increase ROSC in experimental models where adrenaline has been administered during cardiopulmonary resuscitation (CPR) [15–21].

Esmolol is a selective ultrashort-acting β_1_–AR blocking agent producing competitive blockade of β_1_–ARs in both animals and humans[22]. Its short half-life of 9.2 min makes it uniquely suited as a resuscitation drug where heart rhythms and haemodynamic are unstable and shift rapidly. In a recent case series, esmolol given to patients with refractory ventricular fibrillation (VF) was reported to result in higher ROSC rates and improved survival compared to those who did not receive esmolol [23]. All previous experimental studies on β-AR blockers in cardiac arrest have been performed in arrhythmia models [15–21], while the potential effect of antagonizing the detrimental effects of β–AR stimulation by adrenaline is probably most pronounced in ischaemic hearts. Thus, the aim of this randomized, placebo-controlled study was to evaluate the effect of esmolol administered before adrenaline in an ischaemic porcine cardiac arrest model. We used a single dose of esmolol in order to explore a study design that could be easily adapted to a clinical OHCA setting. We hypothesized that a single dose of esmolol administered before adrenaline would increase post-arrest cardiac function. Our primary outcome was post-arrest cardiac output, whereas secondary outcomes included initial resuscitation success, myocardial injury and additional post-arrest cardiac function parameters.

## Methods

### Design

This was a blinded block-randomized placebo-controlled intervention study in pigs to compare initial resuscitation success and post-arrest cardiac function with intravenous esmolol (Brevibloc® 10 mg/ml, Baxter, Deerfield, IL, USA) versus control (9 mg/ml NaCl) during resuscitation with veno-arterial extracorporeal membrane oxygenation (VA-ECMO) following acute myocardial infarction and cardiac arrest. Non-participating personnel had access to the randomization list and prepared the study drug. Investigators remained blinded to randomization until all data were collected and analysed. The study was performed at The Intervention Center, Oslo University Hospital. Equipment used in the experiments is presented in detail in the additional materials.

### Anaesthesia and animal preparation

We included 20 healthy crossbreed Norwegian Landrace pigs (48 kg (range 46–51)) of either sex in the study. The animals were anaesthetized and surgically prepared as previously described in detail [24]. In brief, after fasting overnight aside from free water access, the animals were pre-medicated in the animal facility (Department of Comparative Medicine, University of Oslo) by an intramuscular injection with a mixture of 30 ml ketamine 50 mg/ml (30 mg/kg), 4 ml azaperone 40 mg/ml (3 mg/kg) and 1 ml atropine 1 mg/ml (20 μg/kg) and then transported to the operating theatre. Further anaesthesia was provided with a weight standardized mixture of pentobarbital 4 mg/kg/h, morphine 2 mg/kg/h and midazolam 0.15/kg/h suspended in Ringer`s acetate solution infused at 10 ml/kg/h. Rocuronium was infused at 3 mg/kg/h. The pigs were mechanically ventilated with tidal volume 10 ml/kg, respiratory rate (RR) 18/min, positive end-expiratory pressure (PEEP) 5 mmH_2_O, fraction of inspired oxygen 0.4 and ratio of inspiratory to expiratory time (I:E ratio) 1:2. PaO_2_ and PaCO_2_ were adjusted according to blood gas analyses throughout the experiment.

The surgical preparation included a tracheostomy and placement of a left intraventricular pressure catheter, a carotid arterial pressure catheter and a pulmonary artery (PA) catheter. In addition, ECMO cannulas were positioned through the right internal jugular vein and the left femoral artery [24].

### Experimental protocol (Fig. 1)

After the surgical preparation and a following 30-min stabilization period, pre-arrest (baseline) haemodynamic measurements and cardiac MRI were obtained and blood samples were withdrawn for analyses of serum concentrations of cardiac Troponin T (cTnT) and aspartate transaminase (ASAT).

**Fig. 1:**
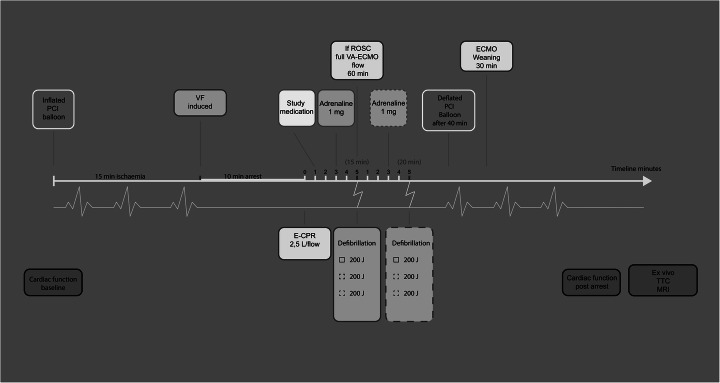
Experimental timeline. Overview of the experiment. *PCI balloon* percutaneous coronary intervention balloon, *VF* ventricular fibrillation, *E-CPR* extracorporeal cardiopulmonary resuscitation, *ROSC* return of spontaneous circulation, *ECMO* extracorporeal membrane oxygenation

The pigs were connected to the Ringer`s acetate primed VA-ECMO circuit with the circuitry set to stand-by and the vascular connections clamped. An intravenous injection of heparin 2 mg/kg followed by 0.5 mg/kg/h infusion was provided to prevent blood clotting in the ECMO circuit. Activated clotting time (ACT) was targeted to ≥ 300 s.

A myocardial infarction was induced by fluoroscopy-guided intravascular balloon occlusion of the proximal coronary circumflex artery. After 15 min of occlusion, VF was induced using a 9 V trans-thoracic current device and confirmed by electrocardiography (ECG) and a rapid aortic arterial pressure drop. The VF was left untreated for 10 min with ventilation on hold. Resuscitation with low-flow (2.5 l/min) VA-ECMO was then initiated and mechanical ventilation restarted with RR set to 10/min. VA-ECMO flow rate was selected to mimic flow generated by optimal CPR. Identical volumes of study drug, (either esmolol 1 mg/kg or NaCl 9 mg/ml—control) were administered 1 min after the initiation of VA-ECMO, 2 min prior to 1 mg adrenaline administration and 4 min prior to the first defibrillation attempt to allow adequate circulation of drugs. The first attempt to terminate VF was thereby initiated 15 min after VF induction using biphasic 200-Joule defibrillation(s) (maximum 3). If the first defibrillation failed, adrenaline was re-administered after 3 min, and defibrillation(s) (maximum three) was repeated 2 min after adrenaline injection. The study protocol allowed a maximum of two 5-min cycles with adrenaline and up to six defibrillations. Pilot experiments suggested resuscitation beyond 10 min of ECMO and six defibrillations to be futile. The balloon in the circumflex artery was deflated after a 40-min occlusion-time.

Successful defibrillation was followed by a 1-h ECMO-support at the maximum achievable ECMO blood flow. During ECMO-support, lower limits of mean arterial pressure (MAP) and pulse pressure were 50 mmHg and 15 mmHg, respectively, sustained if necessary by fluid (max 1 l of Ringer’s acetate) and dobutamine infusions (maximum 5 μg/kg/min). The ECMO-support was reduced by 1/6 of maximum flow every 5 min during a 30-min weaning period in accordance with a standardized weaning protocol. The total VA-ECMO duration was median 100 min for the esmolol group (range 95–110 min) and median 95 min for the placebo group (range 95–103 min). After successful ECMO weaning, dobutamine was discontinued. Post-arrest haemodynamic measurements and cardiac MRI were re-assessed after an additional 30-min stabilization period. Analysis of serum concentrations of cTnT and ASAT were also repeated post-arrest.

The animals were euthanized with an intravenous injection of potassium chloride 1 mmol/kg, morphine 1 mg/kg and pentobarbital 20 mg/kg. Immediately post-mortem, a median sternotomy was performed and the heart was excised, sliced and stained in triphenyl tetrazolium chloride (TTC) to estimate myocardial infarction size.

### Cardiac function assessments

Arterial, pulmonary artery and left ventricular pressures were measured continuously. Maximum and minimum left ventricular pressure pressures (LVP_max_, LVP_min_,) and the related first time-derivate of LVP (LV dP/dt_max_ and dP/dt_min_) were also determined. Left ventricle (LV) function at baseline and post-arrest were assessed by MRI and included measurements of stroke volume (SV), cardiac output (CO = SV × heart rate (HR)), end-diastolic volume (EDV), end-systolic volume (ESV), ejection fraction (EF = SV/EDV), mitral annular plane excursion (MAPSE) and mid-LV radial wall thickening.

Cardiac MRI was performed as previously described in detail [24], and in accordance with the recommendations from the Society for Cardiovascular Magnetic Resonance [25]. Cardiac output was assessed by phase-contrast imaging with blood velocity encoded through-plane images of the mid-ascending aorta, a technique considered to provide robust and accurate cardiac output measurements [24, 26]. The images were analysed using the software Medviso Segment version 2.1 R6005 (http://segment.heiberg.se) [27]. Intra- and inter-observer analyses were performed.

### MRI-assessed infarct size and histological staining

Before euthanasia, each pig received a dose of gadolinium contrast medium to enhance the infarcted area on the ex vivo MRI scans of the heart. A combination of manually drawing and auto-detection tool was used to estimate myocardial infarct size with the software Medviso Segment version 2.1 R6005 [27].

After euthanasia and post-mortem MRI, the left ventricle was excised and cut into approximately 0.5 cm thick slices before staining in tetrazolium chloride (TTC) at 38 °C for 20 min. Infarct size was determined as percentage of the left ventricle [28, 29] using Photoshop CC2017, version 18.01.

### Statistics

Statistical analyses were performed using IBM SPSS Statistics Software version 25. Values are reported as medians with 95% confidence intervals. Groups were compared using Pearson’s chi-squared test or Wilcoxon-Mann-Whitney U test as appropriate. Based on initial pilot and previous experiments, we estimated that post-arrest cardiac output would be 3.3 ± 0.5 l/min in the control group. Assuming cardiac output improved by 15–20% with esmolol (up to 4.0 ± 0.5 l/min), we estimated needing approximately 10 animals in each group with *α* = 0.05 and power 1-β = 0.9.

### Interobserver and intraobserver variability

Four different MRI baseline measurements and two different MRI post-arrest measurements were randomly selected to investigate inter- and intraobserver variability of ejection fraction and cardiac output. The intraclass correlation coefficient of inter- and intraobserver variability for ejection fraction were 0.96 (95% CI 0.7–1.0; *p* < 0.001) and 0.98 (95% CI 0.87–1.0; *p* < 0.001), respectively, and for cardiac output 1.0 and 1.0 (95% CI 0.99–1.0), respectively.

## Results

There were no differences in baseline characteristics between the two groups. The esmolol group consisted of six males and four females with average weight 48 kg (95% CI 47, 51), and placebo group seven males and three females with average weight 48 kg (95% CI 46, 51). Baseline haemodynamic, blood gas values and serum levels of cTNT and ASAT are shown in Table 1.

**Table 1:** Haemodynamic-, MRI-, and blood-gas measurements. Values are expressed as medians with 95% confidence intervals (lower limit, higher limit). Groups are compared using Wilcoxon- Mann-Whitney U test. *MRI* magnetic resonance imaging, *HR* heart rate, *MAP* mean arterial pressure, *MPAP* mean pulmonary artery pressure, *Wedge* pulmonary wedge pressure, *CVP* central venous pressure, *LVP*_*max*_ maximum left ventricle (LV) pressure, *dP/dt*_*max*_ maximum LV pressure first time derivate, *dP/dt*_*min*_ minimum LV pressure first time derivate, *LV ESP* left ventricular end-systolic pressure, *LV EDP* left ventricular end-diastolic pressure, *COphase* cardiac output by cardiac MRI phase-contrast technique, *LV EDV* left ventricular  end-diastolic volume, *LV ESV* left ventricular end-systolic volume, *Wall thickening septum* and *Wall thickening lat* mid-left ventricular radial wall thickening in septum and lateral wall, *MAPSE* mitral annular plane systolic excursion , *LV EF* left ventricular ejection fraction. *SvO*_*2*_ mixed venous oxygen saturation, *ASAT* aspartate transaminase, *cTnT* cardiac troponin T, *TTC* triphenyl tetrazolium chloride tissue-staining, *LV* left ventricle

Variable	Esmolol-group		Control group		*P* value Difference between groups post-arrest
	Baseline	Post-arrest	Baseline	Post-arrest	
	(*n* = 10)	(*n* = 7)	(*n* = 10)	(*n* = 5)	
Haemodynamic variables					
HR (beats/min)	78 (69, 89)	137 (95, 180)	70 (66, 88)	114 (90, 142)	0.3
MAP (mmHg)	96 (78, 112)	75 (62, 88)	92 (80, 103)	79 (60, 84)	0.9
MPAP (mmHg)	22 (18, 25)	21 (15, 28	21 (16, 25)	24 (17, 30)	0.6
Wedge (mmHg)	14 (11, 17)	13 (11, 23)	13 (10, 24)	20 (4, 23)	0.3
CVP (mmHg)	8 (6, 12)	7 (4, 11)	9 (8, 12)	9 (7, 12)	0.4
LVPmax (mmHg)	106 (93, 120)	89 (72, 105)	110 (95, 117)	90 (71, 98)	1.0
LV ESP (mmHg)	62 (53, 80)	52 (41, 58)	65 (53, 75)	57 (41, 62)	0.2
LV EDP (mmHg)	16 (11, 19)	16 (6, 24)	16 (14, 21)	19 (15, 28)	0.2
LV dp/dt_max_ (mmHg/s)	1771 (1248, 2448)	1916 (1322, 3691)	1677 (1349, 1989)	1486 (845, 1870)	0.1
LV dp/dt_min_ (mmHg/s)	− 2339 (− 1756, − 2664)	− 2108 (− 1163, − 2944)	− 2479 (− 1608, − 3624)	− 1586 (− 1375, − 2261)	0.3
MRI measurements					
COphase (l/min)	4.8 (4.2, 5.6)	3.5 (3.3, 3.7)	4.9 (4.6, 5.3)	3.3 (3.2, 3.9)	0.7
LV SV (ml)	61 (55, 69)	27 (18, 39)	69 (62, 75)	31 (20, 36)	0.4
LV EDV (ml)	108 (104, 132)	87 (72, 91)	117 (108, 130)	103 (86, 124)	0.04
LV ESV (ml)	53 (46, 69)	62 (53, 70)	50 (42, 62)	75 (65, 100)	0.03
LV EF (%)	50 (41, 66)	26 (21, 33)	60 (44, 65)	25 (19, 29)	0.5
Wall thickening septum (%)	35 (27, 42)	21 (1, 37)	35 (26, 53)	32 (1, 45)	1.0
Wall thickening lateral (%)	35 (26, 48)	26 (12, 32)	42 (23, 48)	9 (9, 47)	0.4
MAPSE (mm)	14.6 (13.4, 14.6)	6.3 (5.4, 7.5)	13.4 (13.7, 14.3)	6.6 (5.8, 8.6)	1.0
Blood gas analysis					
Hb (g/dl)	8.4 (7.6, 9.8)	9.0 (7.5, 15.2)	8.8 (7.8, 9.3)	9.3 (7.4, 10.0)	1.0
PaO_2_ (kPa)	23.4 (20.9, 28.2)	23.9 (20.1, 34.4	24.9 (20.5, 26.2)	25.3 (23.9, 26.9)	0.9
PaCO_2_ (kPa)	4.7 (4.4, 5.4)	4.6 (2.5, 5.2)	4.8 (4.4, 4.9)	4.5 (4.4, 4.8)	0.9
pH	7.55 (7.52, 7.60)	7.50 (7.50, 7.80)	7.55 (7.50, 7.60)	7.50 (7.50, 7.50)	0.4
Lactate (mmol/l)	1.0 (0.6, 1.5)	2.4 (0.7, 3.7	0.7 (0.7, 1.0)	2.3 (2.1, 2.9)	0.9
SvO_2_ (%)	58 (37, 76)	33 (23, 41)	57 (55, 65)	38 (35, 45)	0.04
Myocardial injury					
ASAT (U/l)	31 (21, 46)	309 (190, 542)	24 (21, 28)	354 (136, 1567)	0.11
cTnT (ng/l)	11 (6, 46)	3864 (2052, 15727)	15 (13, 19)	4014 (1018, 8420)	0.5
Infarct size by MRI (% of LV)	…	20 (18, 32)	…	22 (7, 25)	0.4
Infarct size by TTC (% of LV)	…	20 (16, 30)	…	25 (9, 34)	1.0

### Post-arrest cardiac function

**Fig. 2:**
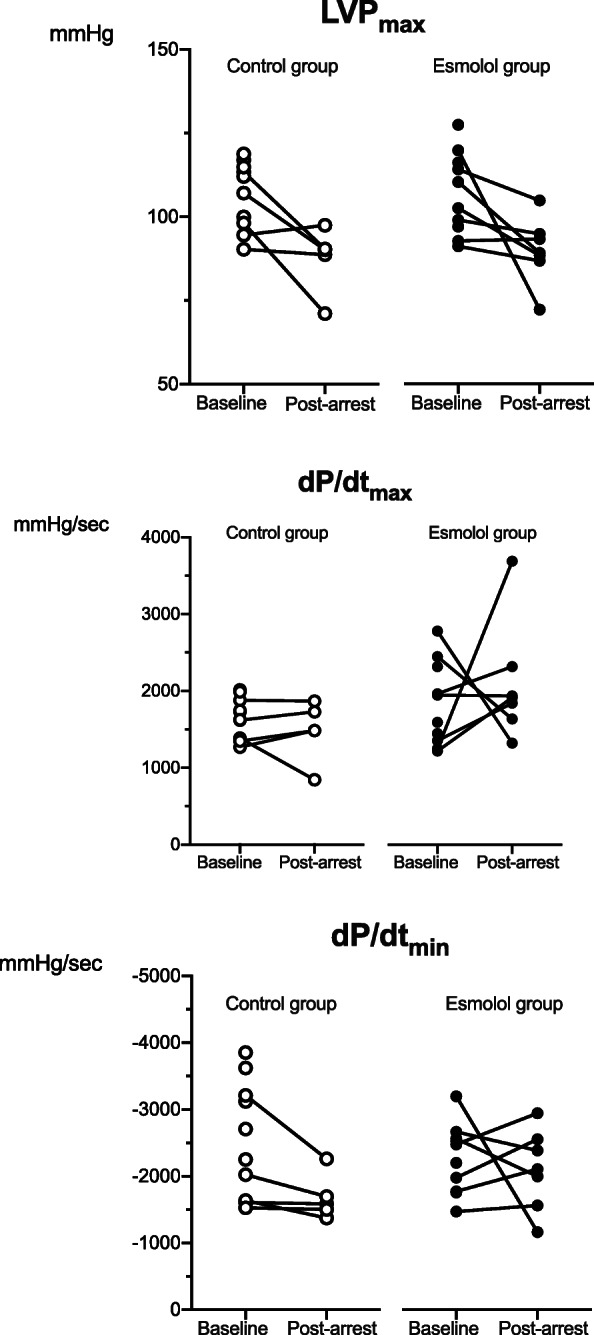
Left ventricle pressure measurements. Left ventricle (LV) pressure measurements at baseline and post-arrest were compared between control and esmolol groups using Pearson’s chi-squared test. There were no significant differences in LVP_max_ (*p* = 1.0), dP/dt_max_ (*p* = 0.1) or dP/dt_min_ (*p* = 0.3). *LVP*_*max*_ maximum left ventricle (LV) pressure, *dP/dt*_*max*_ maximum LV pressure first time derivate or maximum rate of LV pressure increase, *dP/dt*_*min*_ minimum LV pressure first time derivate or maximum rate of LV pressure decrease

**Fig. 3:**
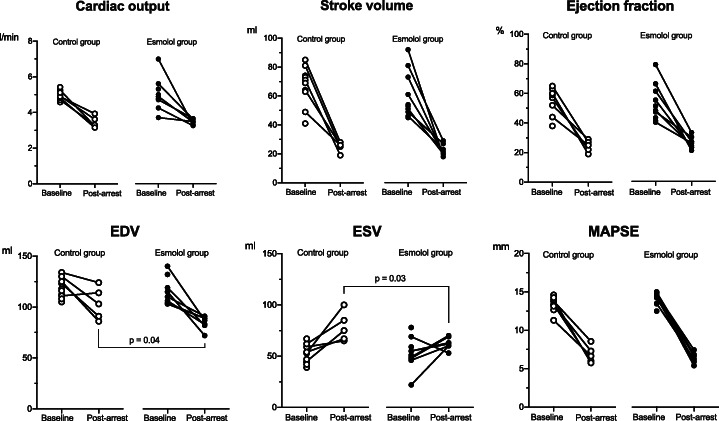
Cardiac MRI measurements. Cardiac magnetic resonance imaging (MRI) measurements of the left ventricle at baseline and post-arrest were compared between control and esmolol groups. Data were compared using the Wilcoxon-Mann-Whitney *U* test. *EDV* left end-diastolic volume, *ESV* left end-systolic volume, *MAPSE* mitral annular plane systolic excursion

All animals had significantly reduced cardiac function post-arrest, but there were no significant differences in our primary outcome, cardiac output, between the esmolol and placebo groups (3.5 (3.3, 3.7) vs. 3.3 (95% CI 3.2, 3.9), respectively, *p* = 0.7). There were no differences in arterial, central venous, pulmonary artery or left ventricular pressures between the two groups post-arrest. In addition, there was no difference in HR or in contraction, expressed as dp/dt_max_, and relaxation, expressed as dp/dt_min_ (Fig. 2, Table 1). Both left ventricular end-systolic and end-diastolic volumes were significantly lower in the esmolol vs the control group; median 62 (95% CI 53, 70) vs. 75 (95% CI 65, 100) ml, *p* = 0.03 and 87 (95% CI 72, 91) vs. 103 (95% CI 86, 124) ml, *p* = 0.04, respectively). Despite differences in ventricular volumes between the two groups, there were no significant differences in any of the other cardiac function parameters. (Fig. 3, Table 1)

### Initial resuscitation

All animals in the esmolol group and 9 of 10 animals in the control group had ROSC (*p* = 0.3), but only 7 of 10 esmolol animals and 5 of 10 control animals were successfully weaned from ECMO (*p* = 0.7). The two groups received similar numbers of defibrillations; median 3 (95% CI 1, 7) vs. 4 (95% CI 1, 9) for esmolol and control groups, respectively, (*p* = 0.9), received the same amount of adrenaline (2 mg for both esmolol and control groups, respectively) and 7 of 10 animals in both groups needed transient post-arrest dobutamine infusions. In addition, 2 of 5 in the control group and 5 of 7 in the esmolol group needed dobutamine infusion during the weaning and stabilization phase, discontinued before post-arrest measurements. Only one animal, in the esmolol group, needed dobutamine infusion throughout the experiment (Table 2).

**Table 2:** Outcome of resuscitation and pharmacological support. Values are expressed as medians with 95% confidence intervals (lower limit, higher limit). Groups are compared using Wilcoxon-Mann-Whitney test *U* test for continuous variables and Pearson’s chi-squared test for categorical variables. *ROSC* Return of spontaneous circulation

Variable	Esmolol-group	Control group	*p* value
	(*n* = 10)	(*n* = 10)	
Number of defibrillations	3 (1, 7)	4 (1, 9)	0.9
Successful defibrillation	10 of 10	9 of 10	0.3
Sustained ROSC	7 of 10	5 of 10	0.7
Adrenaline dose (mg)	2 (1, 2)	2 (1, 2)	1.0
Need for dobutamine post-arrest	5 of 7	2 of 5	0.3
Dobutamine dose (mg)	4.7 (3, 59)	8.5 (3, 14)	1.0

### Post-arrest injury

Infarct size was similar in the two groups as evaluated with both TTC staining and MRI. With TTC staining infarct size was median 20 (95% CI 16, 30) vs. 25 (95% CI 9, 34) % of the left ventricle for esmolol and control groups, respectively, p = 1.0, and with MRI median 20 (95% CI 18, 32) vs. 22 (95% CI 7, 25) % of left ventricle for esmolol and control groups, respectively, *p* = 0.4 (Table 1). There were no differences in markers of myocardial injury (ASAT and cTNT) between the two groups (Table 1). Post-arrest SvO_2_ values were low for both groups, but significantly lower in the esmolol group; median 33 (95% CI 23, 41) vs. 38 (95% CI 35, 45) % for control group, *p* = 0.04) (Table 1). Lactate values were within the upper normal range for both groups; median 2.4 (95% CI 0.7, 3.7) vs. 2.3 (95% CI 2.1, 2.9) mmol/l for esmolol and control groups, respectively, *p* = 0.9 (Table 2).

## Discussion

This study used an acute ischaemic porcine cardiac arrest model to investigate whether a single dose of esmolol given during the initial phase of resuscitation, and prior to administration of adrenaline, could improve initial resuscitation success and post-arrest cardiac function. Although 70% of the animals were successfully resuscitated in the esmolol group compared to 50% in the control group, the study was aimed and powered to evaluate post-arrest cardiac output, not survival outcomes, and therefore unable to demonstrate a statistical difference on resuscitation success. While we observed statistically significant differences in end-systolic and diastolic volumes and mixed central venous oxygen saturation, we were unable to demonstrate that esmolol protects against post-arrest cardiac dysfunction defined as post-arrest cardiac output. As very many parameters were compared between the two groups, there is also an increasing risk that some of these parameters will be statistically different by pure chance, and that the statistically significant differences observed may simply be related to the multiple comparisons between the two groups. Due to the exploratory design of the study, statistical correction for multiple comparisons was not performed.

Pre-arrest β-AR-blocker use has been associated with improved survival in patients with VF [30], and patients with implanted cardioverter defibrillators (ICDs) on previous β-AR blocker medication have been observed to experience fewer ICD defibrillations compared to those not using β-AR -blockers [31]. Additionally, two recent case series both reported better outcomes for a group of refractory VF cardiac arrest patients which received esmolol compared to a group that did not [23, 32]. Although the authors attempted to match patients who received esmolol to similar patients that did not, there is obvious risk of bias and unrecognized confounding in such non-randomized designs. Nonetheless, these observations are interesting and warrant further exploration.

Although generally encouraging, previous experimental studies on esmolol during cardiac arrest have been divergent. Some studies have observed improved ROSC rates with fewer defibrillations in animals given esmolol in addition to adrenaline compared to animals given adrenaline alone [14, 15, 18, 20, 33], whereas other studies have been unable to demonstrate a clear benefit from esmolol during initial resuscitation [34–37]. Similarly, some studies have reported impressive improvements in post-arrest cardiac function with esmolol [14, 16, 18], while other studies have failed to observe any differences in relevant haemodynamic variables [34–37]. These conflicting results reflect the complexities of designing clinically relevant and robust experimental models, as well as raise the question whether (a) esmolol might not be a useful resuscitation drug or (b) we have not elucidated optimal timing, dose or form of administration.

If esmolol is beneficial during resuscitation from cardiac arrest, continuous infusion strategies where esmolol may be titrated to specific individual targets, (e.g. heart rate) could potentially be more effective than simpler bolus strategies. However, such strategies would effectively exclude use in ordinary prehospital settings where most cardiac arrests occur. As such, an effective bolus strategy has the greatest potential to translate from the experimental laboratory setting into clinical practice. The studies previously demonstrating benefits from esmolol during resuscitation in experimental animal models have used single doses of 0.3–1 mg/kg [14–16, 18, 20, 33] administered at the beginning of the resuscitation effort, comparable to the present 1 mg/kg dose. However, a previous dose-exploring study evaluating cardioprotective effects of esmolol during cardiac surgery suggested that 0.25 mg/kg was superior to 0.5 mg/kg and that using higher doses might cause sustained reduction in cardiac function probably related to the negative inotropic effect [38]. We can only speculate whether reduced end-systolic and diastolic volumes and mixed central venous oxygen saturation in the esmolol group reflects the high esmolol dose used. On the other hand, the trend towards more frequent and prolonged need for dobutamine support in the esmolol group could also be related to a higher total ß adrenergic load resulting in the trend towards higher heart rates in the esmolol group.

Many promising drugs have failed to successfully translate from the laboratory to human trials, and the lack of clinically relevant models is considered to be an important limitation to preclinical research [39]. Clinical cardiac arrest research on the other hand is often limited by the lack of specificity, as peri-arrest factors are often unknown during resuscitation. Clinical studies inherently include patient cohorts consisting of both patients who are easily resuscitated by early defibrillation and patients who are well beyond resuscitation, in addition to the sub-group of patients where an intervention has the potential to improve outcome. Another typical challenge for clinical studies is the heterogeneity of causes of arrest among included patients, resulting in different effects of interventions tested [40]. As an example, interventions might improve haemodynamic and outcome in patients with myocardial infarction, but not in patients with cardiac arrhythmias of non-ischemic origin. Thus, a stronger linkage between specific clinical cardiac arrest phenotypes and tailored preclinical models may improve the relevance of preclinical cardiac arrest studies [41].

### Limitations

There are several limitations worth mentioning. Firstly, as with any animal experimental model, it will never fully replicate the heterogeneous clinical setting. Although we induced acute myocardial infarction with our ischaemic model, animals were young and otherwise healthy prior to our experiments. Similarly, initiation of low-flow VA-ECMO does not represent the physiology during manual CPR because chest compressions provide a different effect on vital perfusion through their effect on intrathoracic pressure, venous pressure and pulsatile flow, thereby affecting the haemodynamic response of esmolol and epinephrine. However pilot testing indicated we would be unable to resuscitate all animals with prolonged arrests using manual or mechanical CPR, and we therefore prioritized a resuscitation strategy that would enable initial resuscitation with significant post-arrest myocardial dysfunction instead of more commonly used clinical strategies. Anatomical differences and variations in balloon placement in the coronary arteries might also confound infarct size and post-arrest cardiac function, although great care was taken to standardize procedures and minimize these effects. Additionally, administration of a vasopressor such as noradrenaline or vasopressin alone has been tested before [42] , and could perhaps have added insight as a third group in our study. Finally, although we included ten animals in each group, fewer animals than expected were resuscitated leading to an underpowered evaluation of post-arrest cardiac output. However, since there was no clear signal that the “single-dose esmolol” was effective in improving post-arrest myocardial function, we believe it is unlikely that additional experiments would add significant insight. We propose evaluating repeated doses or continuous infusion of esmolol might be more promising avenues to pursue in future experimental exploration of esmolol.

## Conclusions

We observed similar post-arrest cardiac output with and without a single dose of esmolol prior to adrenaline administration during low-flow VA-ECMO in an ischaemic cardiac arrest pig model. While not sufficiently powered to provide a definitive answer, the similarity between the two groups indicates other avenues or models should be encouraged.

## Electronic Supplementary Material

Supplementary material, approximately 16.0 KB.

## Data Availability

The datasets used and analysed during the current study are available from the corresponding author on reasonable request.

## Abbreviations

COphase: cardiac output with cardiac MRI phase-contrast technique
CVP: central venous pressure
HR: heart rate measured with cardiac MRI
LV dP/dt_max_ and dP/dt_min_: maximum and minimum LVP time derivates
LV EDP: left ventricular end-diastolic pressure
LV EDV: left ventricular end-diastolic volume
LV EF: left ventricular ejection fraction
LV ESP: left ventricular end-systolic pressure
LV ESV: left ventricular end-systolic volume
LVP_max_: left ventricular systolic pressure maximum
MAP: mean arterial pressure
MAPSE: mitral annular plane systolic excursion
MPAP: mean pulmonary arterial pressure
Wall thickening septum and Wall thickening lat: mid left ventricular radial wall thickening in septum and lateral wall
Wedge: pulmonary arterial wedge pressure
