# Supplementary material for: Esmolol for cardioprotection during resuscitation with adrenaline in an ischaemic porcine cardiac arrest model
Source: Intensive Care Med Exp. 2019 Dec 4;7:65. doi: 10.1186/s40635-019-0279-5 (PMC6892997; doi:10.1186/s40635-019-0279-5)
Supplement: Supplementary file 1 — Supplementary material, approximately 16.0 KB. [file 40635_2019_279_MOESM1_ESM.docx]

| **Table 1 Equipment** | | | | |
| --- | --- | --- | --- | --- |
| **Accessories** | **Name** | **Company** | **Location** | **Country** |
| Ventilator | Leon Plus | Heinen & Lövenstein | Bad Erms | Germany |
| Ventilator in MRI | Fabius MRI | Drägerverk AG & Co | Lübeck | Germany |
| Left ventricular pressure catheter | MPR-500 | Millar Instruments | Houston, TX | USA |
| Carotid arterial pressure catheter | Radifocusintroducer II 8Fr | Terumo Europe | Leuven | Belgium |
| Pulmonary artery catheter | Swan-Ganz CCO | Edwards Lifesciences | Irvine, CA | USA |
| Pressure transducer software | Mikrosound presenter 18.11.2011 | Vestfold university college | Tønsberg | Norway |
| Pulmonary artery monitor | Vigilance II Monitor | Edwards Lifesciences | Irvine, CA | USA |
| Defibrillator | ZOLL Medical Corporation | ZOLL Medical Corporation | Chelmsford, MA | USA |
| Fluoroscopy-guided intravascular balloon | TREK RX Coronary Dilatation | Abbot Vascular | Santa Clara, CA | USA |
| Automated Coagulation Timer System | Hemochron Jr Signature+ | ITC | Edison, NJ | USA |
| Tetrazolium chloride | TTC 1% phosphate buffered saline | Sigma Aldrich | St.Louis, MO | USA |
| Tissue staining/TTC imaging software | Photoshop CC2017 Version 18.01 | Adobe Systems Software Ltd |  | Ireland |
| Data analysis software | Graphpad prism 8 | GraphPad Software | La Jolla, CA | USA |
| Statistics software | IBM SPSS version 25 | SPSS | Chicago, IL | USA |
| Graphpad prism | Version 8 | GraphPad Software | La Jolla, CA | USA |
| **ECMO circuit** | **Name** | **Company** | **Location** | **Country** |
| Arterial cannulae | DLP Femoral 14Fr | Medtronic Inc | Minneapolis, MN | USA |
| Venous cannulae | DLP Jugular 21Fr | Medtronic Inc | Minneapolis, MN | USA |
| Centrifugal pump | Biopump + BPX-80 | Medtronic Inc | Minneapolis, MN | USA |
| Oxygenator | Affinity fusion | Medtronic Inc | Minneapolis, MN | USA |
| Console | Biomedicus 550 Bio-console | Medtronic Inc | Minneapolis, MN | USA |
| Flow transducer | BioProbe TX50 | Medtronic Inc | Minneapolis, MN | USA |
| Heat-exchanger | Stöckert Heater-Cooler System 3T | Sorin Group | Milano | Italy |
| Oxygen/air mixer | Fusion | Medtronic Inc | Minneapolis, MN | USA |
| **MRI** | **Name** | **Company** | **Location** | **Country** |
| MRI scanner w/software | Philips Ingenia 3 Tesla. MR software release 5.1 | Philips Medical Systems | DA Best | Netherland |
| MRI cine/phase contrast image analysis | Segment version | Medvisio AB | Lund | Sweden |

Equipment used in the experiment.
